# Cognitive control and meta-control in dual-task coordination

**DOI:** 10.3758/s13423-023-02427-7

**Published:** 2023-12-12

**Authors:** Tilo Strobach

**Affiliations:** Department of Psychology, Medical School Hamburg, Am Kaiserkai 1, 20457 Hamburg, Germany

**Keywords:** Dual tasks, Task order, Dual-task coordination, Coordination adjustment, Psychological refractory period paradigm

## Abstract

When two tasks are presented simultaneously or in close succession, such as in the overlapping task paradigm of the psychological refractory period, dual-task performance on those tasks is usually impaired compared with separate single-task performance. Numerous theories explain these emerging dual-task costs in terms of the existence of capacity limitations in the constituent component tasks. The current paper proposes active dual-task coordination processes that work on the scheduling of these capacity-limited processes. Further, there are recent findings that point to a meta-cognitive control level in addition to these active coordination processes. This additional level’s responsibility is to adjust the dual-task coordination of capacity-limited stages (i.e., coordination adjustment). I review evidence focusing on the existence of dual-task coordination processes and processes of coordination adjustment. The remainder of the paper elaborates on preliminary findings and points to the separability of these sets of processes, which is a key assumption of the framework of dual-task coordination adjustment.

## Introduction

Our real-world experience is that performing two tasks simultaneously compromises performance in one or both of these tasks. That is, there are dual-task costs that manifest in an increase in errors and/or the time needed to perform the two tasks, as compared with their separate performance in single-task situations. Studies in the context of the overlapping task paradigm of the psychological refractory period (PRP) investigated these costs through the presentation of two stimuli and a variable interval between their onsets (stimulus-onset asynchrony; SOA), and participants have to make choice reaction time (RT) responses to these stimuli. The typical finding is that RTs to the second (task) stimulus (RT2) increase with decreasing SOA (e.g., Pashler, 1994; Schubert, 2008; Strobach et al., 2015); this RT2 pattern is referred to as the PRP effect, reflecting dual task costs. The popularity of the PRP paradigm stems from the general robustness of the PRP effect in a variety of situations and contexts (i.e., the PRP effect appears under numerous conditions), but this effect is also sensitive to modulations across these conditions (Pashler & Johnston, 1989, 1998). This sensitivity makes the PRP paradigm a popular tool to investigate capacity limitations and attentional control of two simultaneous tasks in the human cognitive system.

**Fig. 1:**
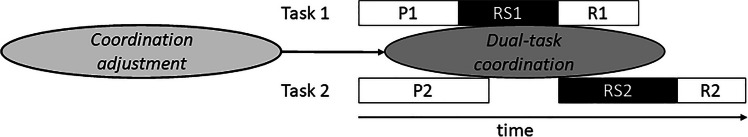
Illustration of the framework of dual-task coordination adjustment (DTCA framework). *Note.* Illustration of the hypothetical time relation of processing stages in the component Tasks 1 and 2, when presented in a dual-task situation with stimulus-onset asynchrony (SOA) = 0 ms. As illustrated, there is a capacity limitation within the component tasks that does not allow simultaneous processing of the associated stages (i.e., the response selection stage; Level a). Processes of dual-task coordination are associated with the coordination of the two independent task processing streams in dual-task situations, and they are conceptualized as an element that is distinct from the component tasks (Level b). Additional components of coordination adjustment modulate this coordination (Level c). Note that the illustrated locations of dual-task coordination and coordination adjustments are not explicitly related to the temporal relations between these components. P1 and P2 indicate the perception stages; RS1 and RS2 indicate the central response-selection stages (including bottleneck characteristics); R1 and R2 indicate the response stages

The PRP effect has been attributed to capacity limitation models, assuming that two tasks can simultaneously share processing resources so that neither task in dual tasks is performed as quickly as it would be performed in single tasks (e.g., Hazeltine et al., 2006; Meyer & Kieras, 1997; J. Miller & Durst, 2015; J. Miller et al., 2009; Mittelstädt & Miller, 2017). The specific strategy of a full distribution of processing resources to one component task and no such resources shared with the other task is consistent with the assumptions of the central bottleneck model. According to this model, a full distribution of processing resources to one task is explained with a structural and unavoidable processing bottleneck limitation. This bottleneck limitation prevents the two tasks from being performed in parallel. As illustrated in Fig. 1, this bottleneck limitation is basically located at a central processing stage between stimulus perception and response execution—namely, the response selection stage (e.g., Pashler, 1994; Welford, 1952, 1980). Irrespective of its nature (i.e., strategic capacity limitation or structural bottleneck limitation), the notion of a processing-limited central response selection accounts for the PRP effect by assuming that, if the two tasks are presented with short SOAs between Task Stimulus 1 and Task Stimulus 2, response selection for the two tasks operates serially while the initial perception and final response execution stages of the two tasks can basically operate in parallel (however, see Bratzke et al., 2009; de Jong, 1993, for additional bottleneck limitations on response stages). In other words, response selection for the second task is postponed until the end of this stage in the first task and until the first task has left the bottleneck stage; this postponement increases with decreasing SOA. Importantly, recent studies have provided evidence that the scheduling of bottleneck access for Task 1 and Task 2 in a PRP situation is not a passive mechanism but involves processes of active task coordination (de Jong, 1995; Hendrich et al., 2012; Kamienkowski et al., 2011; Meyer & Kieras, 1997; Sigman & Dehaene, 2006; Töllner et al., 2012). As illustrated in Fig. 1, this active task coordination is conceptualized as an element that is distinct from the component tasks and is typically associated with the coordination of two independent and overlapping task processing streams in dual-task situations (Hirst et al., 1980).

The central aim of this review is to link active dual-task coordination (DTC) processes to new findings about their situational adjustment. This review builds on the hypothesis that DTC processes and the processes of their adjustment underlie different mechanisms. If there is conclusive evidence for such a difference, this would be consistent with the assumption of separable types of processes of DTC and the adjustment of these coordination processes. This question of the review promises to investigate the existence of a “coordination adjustment” homunculus—an agent responsible for cognitive control, as it is hotly debated in task-switching situations and situations of conflict processing (Braem et al., 2019; Logan & Bundesen, 2003; Schmidt et al., 2020)—in dual-task situations (Logan & Gordon, 2001).

A review of this aspect of dual tasks is needed and lacking in the current review literature. Recent reviews such as Koch et al. (2018) or Strobach et al. (2020) are partly outdated and, more importantly, have a different focus. These reviews might be outdated because the dual-task research field is a very active field, constantly producing new findings. This can be illustrated by searching for “dual tasks” in the PsycInfo database, which documented 1,697 and 1,074 new studies since 2018 and 2020, respectively (by October 6, 2023), when these previous reviews were published. Further, when looking at the foci of the different reviews, Koch et al. (2018) provide a fairly extensive review of the dual-task literature and integrate it with task switching in terms of flexibility and plasticity issues. Strobach et al. (2020) reviews the literature on dual-task practice, speaking to the practice-related improvement of DTC. In particular, this latter review focuses on the very specific allocation and scheduling hypothesis, where dual-task practice induces participants to adopt a strategy of coordinating two simultaneous tasks, which enables them to develop task coordination skills for improved cognitive resource allocation and scheduling. However, the present review builds on the former ones and goes beyond them with a novel approach focused on meta-control in dual tasks. The critical issue of the timely review, then, is how control and meta-control can be separated empirically, and a focused review on this issue is welcome.

## ﻿The framework of dual-task coordination adjustment

As previously stated, active DTC processes regulate the scheduling of capacity-limited processing stages within component tasks (Fig. 1). Several models of dual-task performance generally discuss those coordination processes. For example, the executive-process interactive control (EPIC) architecture (Meyer & Kieras, 1997) assumes dual-task performance involves coordination of processes (e.g., temporarily locking out processing for one task to avoid conflict in processing for another task). The executive control theory of visual attention (ECTVA; Logan & Gordon, 2001) assumes the strategic setting of component-task parameters to enable dual-task performance that minimizes crosstalk. Adaptive control of thought-rational (ACT-R) models of dual-task performance (e.g., Anderson et al., 2005; Byrne & Anderson, 2001) characterize how component-task processes can be organized within an integrated cognitive architecture. Subsequent developments such as threaded cognition (e.g., Salvucci & Taatgen, 2008) built on that earlier work to show how dual tasks can be coordinated as distinct “threads” of information processing.

Although these models make very detailed mechanistic and/or computational assumptions about dual-task performance, they rarely point to a cognitive control level in addition to the active DTC processes (one of the rare exceptions is the EPIC model, which proposes the daring and cautious adjustment of DTC; Meyer & Kieras, 1997). ECTVA even explicitly states that “The whole [i.e., the dual-task situation] is more than the sum of its parts [i.e., the component tasks] but not much more” (Logan & Gordon, 2001, p. 394). The additional level’s responsibility is to adjust DTC. Figure 1 illustrates this cognitive control level (i.e., coordination adjustment) in the framework of dual-task coordination adjustment (DTCA). Note that this framework basically structures the literature according to three levels: (a) component-task processes that enable the performance of each individual task; (b) DTC processes that schedule limited-capacity lower-level processing stages; and (c) DTCA processes that implement cognitive control to adjust coordination. Table 1 provides a list of key papers on Levels a, b, and c, that is, component tasks, DTC, and DTCA, respectively.

**Table 1:** Lists of exemplary papers for the three proposed theoretical levels of control in dual tasks (i.e., component tasks, dual-task coordination, and coordination adjustment) from nonpractice contexts and from practice contexts

Component tasks	
Nonpractice context (reviews)	Practice context
• Meyer & Kieras, (1997) • Koch et al., (2018) • Pashler, (1994) • Schubert, (2008)	• Ahissar et al., (2001) • Anderson et al., (2005) • Dux et al., (2009) • Garner et al., (2014) • Kamienkowski et al., (2011) • Ruthruff et al., (2001) • Ruthruff et al., (2006) • Sangals et al., (2007) • Strobach et al., (2013)) • Van Selst et al., (1999)
Dual-task coordination	
Nonpractice context	Practice context
• de Jong (1995) • Hendrich et al., (2012) • Hirsch et al., (2017) • HirschKoch et al., (2018) • Hirsch et al., (2021)) • Kübler et al., (2018) • Kübler et al., (2022a) • Kübler et al., (2022b) • Leonhard et al., (2011) • Luria & Meiran, (2003) • Otermans et al., (2022) • Ruiz Fernández et al., (2011) • Sigman & Dehaene, 2006) • Szameitat et al., (2006) • Szameitat et al., (2002)	• Hirst et al., (1980) • Kramer et al., (1995) • Liepelt et al., (2011) • Schubert et al., (2017) • Schubert, & Strobach et al., (2018) • Strobach et al., (2012a) • Strobach et al., (2012b)
Coordination adjustment	
Nonpractice context	Practice context
• Brüning & Manzey, (2018) • Brüning et al., (2020) • Strobach et al., (2021) • Strobach et al., (2023) • Strobach & Wendt, (2022)	• Orscheschek et al., (2019) • Strobach et al., (2014b)

The component task level in nonpractice contexts is mainly represented by reviews on this issue. Papers are indicated by their full list of authors and can be found in the reference list of the present review

In this paper, I summarize a set of findings that demonstrate the existence of active scheduling of capacity-limited processes and DTC mechanisms. In this context, I outline approaches that demonstrate the independence of processing the individual component tasks (Level a) from DTC mechanisms (Level b) (see section Characteristics of Dual-Task Coordination Processes). I then go on to introduce empirical findings, demonstrating the level of dual-task cognitive control (Level c), adjusting DTC (see section Characteristics of Coordination Adjustment).

## Characteristics o﻿f dual-task coordination processes

DTC is typically associated with the coordination of two simultaneous task processing streams. The first key assumption of the DTCA framework is that the processing streams of the individual component tasks that comprise a dual task (Level a) are separated from the processes related to active coordination of these processing streams (i.e., DTC on Level b). There are several approaches separating processes within the component tasks from processes of DTC beyond the component tasks. These approaches stem from the literature (1) on dual-task practice and (2) on dual-task processing without practice components.

### App﻿roaches from the dual-task practice literature

According to a first approach, the literature assumes that there is an optimization of dual-task performance with practice. While some components of this optimization are explained with improvements within the component tasks (e.g., Ahissar et al., 2001; Anderson et al., 2005; Dux et al., 2009; Garner et al., 2014; Kamienkowski et al., 2011; Ruthruff et al., 2001, 2006; Sangals et al., 2007; Strobach et al., 2013; Van Selst et al., 1999), there are additional components of dual-task optimization that are independent from those changes within these tasks. These components are associated with the optimization of DTC skills, allowing for a practice-related improvement of the coordination of two simultaneously presented and overlapping component tasks (Kramer et al., 1995; Maquestiaux et al., 2004). In more detail, I consider these skills are based on mechanisms that efficiently control and schedule two simultaneously ongoing task streams. The nature of these improved task coordination skills could be specified by two theoretical hypotheses (Hirst et al., 1980; Kramer et al., 1995). First, task coordination skills are acquired during dual-task practice (with simultaneously practiced tasks) but not during single-task practice (with tasks practiced separately). In particular, while dual-task practice leads to a more efficient coordination of two simultaneously performed task streams, the mere practice of single tasks does not. Second, once acquired, improved task coordination skills are independent from the practiced task situation. Consequently, task coordination skills acquired in a particular dual-task situation are transferable to other unpracticed situations. Testing these hypotheses is essential to demonstrate the separation of DTC from the component tasks and the general-purpose nature of these DTC skills.

**Fig. 2:**
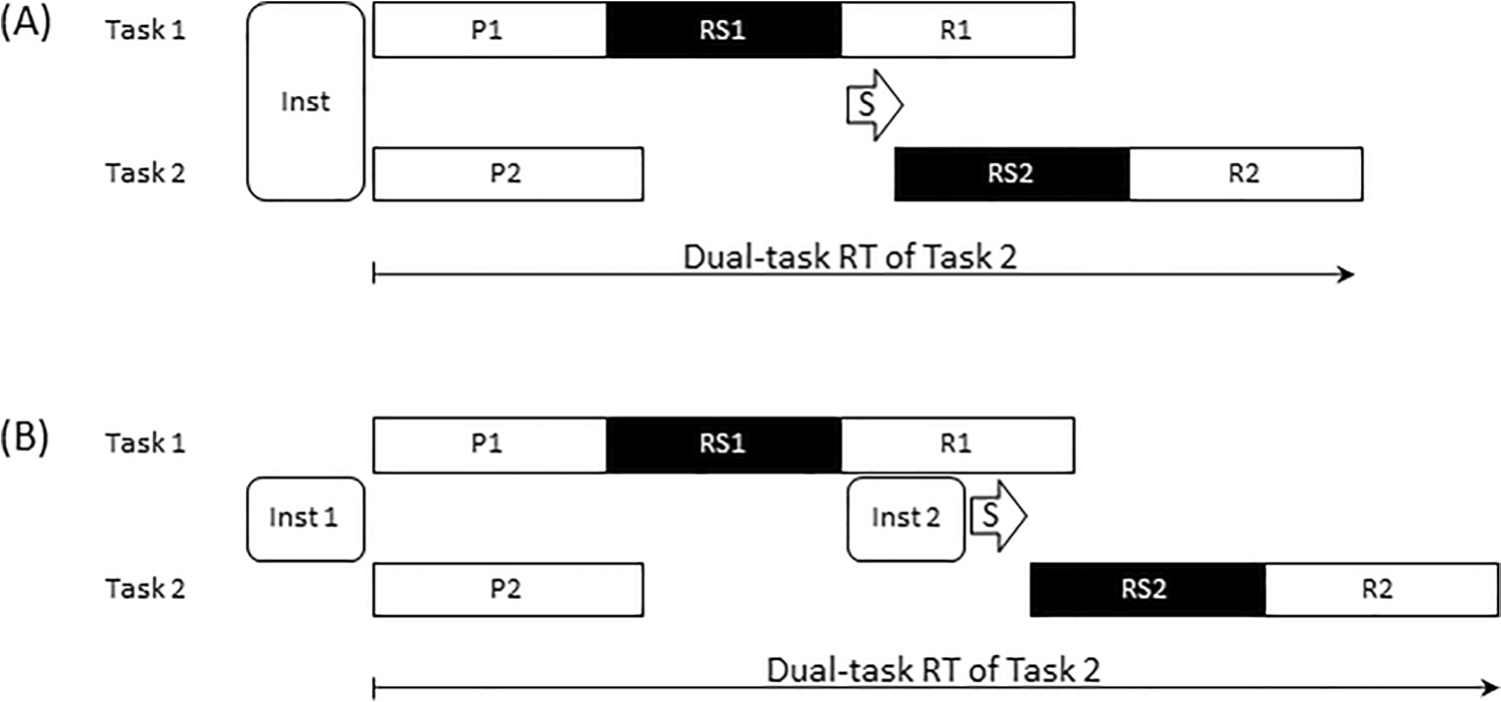
Illustration of the hypothetical time relation of processing stages in a first task (Task 1) and a second task (Task 2) according to the efficient task instantiation model. *Note.* Panel (**A**): Hypothetical time relation of dual-task processing at the end of *dual-task practice* leading to efficient instantiation of the information of two tasks before the start of a dual-task trial (Inst) and to short dual-task RT in Task 2 because of a short switch from RS1 to RS2. Panel (**B**): Hypothetical time relation of dual-task processing at the end of *single-task practice* not leading to efficient instantiation of the information of two tasks at the onset of a dual-task trial (Inst 1) but to an additional instantiation process (Inst 2) after the completion of RS1 and before RS2 and to long dual-task RTs in Task 2. P1 and P2 indicate the perception stages; RS1 and RS2 indicate the central response-selection stages (including bottleneck characteristics); R1 and R2 indicate the response stages. Inst: instantiation of task information; Inst 1: first instantiation of task information; Inst 2: second instantiation of task information; S: switching between component tasks after the completion of RS1 and before the start of RS2

To test these hypotheses related to the optimization of DTC skills, Liepelt et al. (2011) and Strobach et al. (2012b) compared the dual-task performance of two groups of participants, experiencing different types of practice with a visual task and an auditory task. While (1) dual-task practice included intermixed presentations of both tasks in single tasks and dual tasks (including a SOA of 0 ms) in dual-task blocks and separate presentations of both tasks in single-task blocks (see also Hazeltine et al., 2006; Schumacher et al., 2001), (2) pure single-task practice included the exclusive presentation of the visual and auditory tasks in separate single-task blocks. In fact, after six sessions of dual-task practice, dual-task performance in the seventh test session improved when compared with the dual-task performance after six sessions of single-task practice. In detail, this improvement was exclusively demonstrated by reduced dual-task RTs in the auditory task, while there was no such evidence in the visual task. The auditory task and the visual task are typically performed second and first, respectively (see also Hartley et al., 2011; Schumacher et al., 2001; Strobach et al., 2012c, 2012d; Tombu & Jolicœur, 2004). The second responses in the auditory task and the first ones in the visual task are potentially also consistent with the order of processing stimulus–response selection in both component tasks in dual tasks, indicating a second auditory response selection (Task 2) and a first visual response selection (Task 1; Ruthruff et al., 2003). However, as illustrated in Fig. 2, this auditory Task 2 response selection might start more efficiently (and therefore earlier) after dual-task practice in contrast to the effects of single-task practice, which could explain the exclusive Task 2 advantage after the former practice type.

In their efficient task instantiation model, Schubert and Strobach (2018) assumed that this particular advantage after dual-task practice is related to a more rapid switch between component tasks due to efficient instantiation of relevant task information in working memory at the onset of a dual-task trial of the overlapping task paradigm (Fig. 2). This instantiation in working memory primarily involves task-set information of both tasks that constitute a dual task, including their task-relevant information of stimulus–response mapping rules. Importantly, this does not mean that task sets are somehow integrated (i.e., task integration; Ruthruff et al., 2001, 2006), but instantiation means that tasks are represented separately but at the same time in working memory. In general, these findings demonstrate that DTC skills are acquired during dual-task practice but not during single-task practice, and the efficient task instantiation model represents one potential mechanism for this dual-task practice advantage.

These findings also demonstrate a first link between working memory and dual tasking. The acquisition of DTC skills takes into account that the capacity of working memory is limited. Consequently, capacity limitations of the working memory may expose a burden for the success of practice-related changes in task processing (Allen, 2022; Cowan, 2001; Cowan et al., 2005; G. A. Miller, 1956). In more detail, an efficient instantiation of task-set information may be affected by the processing load that is exposed on the system due to (A) the number of task stimulus–response mappings and (B) their compatibility relations in a dual-task situation (see Maquestiaux et al., 2004). It might be the case that under certain conditions, the number of stimulus–response mappings and the task compatibility demands may exceed the available working memory capacity, and this may prevent the cognitive system from instantiating two tasks efficiently even after dual-task practice (see also Huestegge & Koch, 2010). Consistently, a substantial increase in the number of stimulus–response mappings affected and eliminated the dual-task practice advantage, while an increase in compatibility did not affect this advantage; this advantage was similarly evident under rather compatible and incompatible conditions. This perspective widens current views on practice-related changes in dual-task processing because it combines assumptions about the processing architecture during dual-task processing with assumptions about the underlying working memory capabilities of the cognitive system (Strobach et al., 2014a).

The practice-related advantage in the auditory task as described by Liepelt et al. (Liepelt et al., 2011; Strobach et al., 2012a) was not only evident in a dual-task situation that was identical to the practiced situation. This advantage was also evident in dual-task situations that (1) included the practiced visual task and a new, unpracticed auditory task; (2) included a new, unpracticed visual task and the practiced auditory task; and (3) included a new, unpracticed visual task and a new, unpracticed auditory tasks (Schubert et al., 2017). Importantly, the findings of these dual-task situations (1), (2), and (3) demonstrate the transferability of task coordination skills to structurally similar dual tasks (i.e., so-called near-transfer effects; see also Strobach et al., 2012a, for rather far-transfer effects between structurally different task situations). In sum, dual-task practice mechanisms are associated with the acquisition and transfer of improved task coordination skills (Hirst et al., 1980; Kamienkowski et al., 2011; Kramer et al., 1995), enabling the optimized and efficient processing of two simultaneous task streams. These skills are independent from processes within the component tasks and thus generally justify the separation of DTC processes (Level b) from the component task processing streams (Level a), as proposed by the DTCA framework.

### ﻿Approaches from the nonpractice dual-task literature

According to a nonpractice approach to clarify the separation between processes within the component tasks (Level a) and processes of DTC (Level b) in the context of the DTCA framework, the regulation of task order in dual tasks has been investigated in a specific version of the PRP paradigm (Kübler et al., 2018; Luria & Meiran, 2003; Sigman & Dehaene, 2006). In this version, the presentation order of both stimuli varies randomly from trial to trial, and participants are instructed to execute two choice RT tasks according to the order of stimulus presentation, resulting in repetitions of the same task order (i.e., repetition-order trials) and switches between different task orders (i.e., switch-order trials) in successive trials. By applying this paradigm, two potential mechanisms of task-order regulation have been identified. One possibility to characterize order control is that the limited processing stage is simply recruited by the stimuli on a first-come, first-served basis, so that the order in which the tasks are handled is determined by which of the two task stimuli arrives at the limitation first; recent studies provided empirical evidence that is consistent with the predictions of this first-come, first-served mode (Hendrich et al., 2012; Leonhard et al., 2011; Ruiz Fernández et al., 2011; Sigman & Dehaene, 2006; Strobach et al., 2018).

The second possible mechanism of task-order regulation assumes that the order in which the tasks are performed might be additionally controlled by DTC processes (i.e., task-order coordination processes; de Jong, 2000; Schubert, 2008). First evidence of the involvement of task-order coordination in dual tasks comes from fMRI studies demonstrating increased dorsolateral prefrontal activity in dual-task blocks with trials of randomly varying task orders in contrast to the activity in blocks with a constant and predictable task order (Szameitat et al., 2002). In more fine-grained analyses, the activity was particularly increased in switch-order trials in comparison to the activity in repetition-order trials (Szameitat et al., 2006). Further analyses showed that RTs and error rates are increased in switch-order versus repetition-order trials; I refer to this increase as *order switch costs*.

These order switch costs might be the first evidence of the involvement of task-order coordination in dual tasks. However, the mere presence of increased dorsolateral prefrontal activity during switch-order trials does not necessarily imply the presence of a coordination process but could instead reflect passive, expectation-related effects, signaling conflicts. In addition, increased RTs and error rates in switch- versus repetition-order trials can result from violations of expectations for task sequences (Muhle-Karbe et al., 2018) or higher-order binding effects (Frings et al., 2020). Given the example of higher-order binding effects, features of the stimulus environment, the order of these stimuli, responses, and response orders in that environment, as well as subsequent response effects, might be integrated into event files (Hommel, 2004). The repetition of a specific order of stimulus modalities improves the retrieval of an episode, including this order, improving performance in repetition-order trials. In contrast, a switch between specific orders impairs the retrieval of an episode, including this order, and thus impairs the performance in switch-order trials.

Because of these assumptions from theories on passive, expectation-related effects, several studies aimed at characterizing the underlying active DTC mechanisms (Level b in the DTCA framework) that lead to order switch costs. One mechanism explains these costs by assuming task-order representations (Kübler et al., 2018). According to the assumption of task-order representations, in repetition-order trials, the task-order set (representing the task order in a dual-task trial) of the previous trial can be applied in the following trial. However, in switch-order trials, a new task-order set requires its implementation. That means the old task-order set of the previous trial needs to be inhibited and/or the new task-order set of the following trial needs to be activated due to DTC processes.

In alternative investigations on DTC processes (i.e., Level b), Hirsch et al. (2021) applied a paradigm in which two tasks are performed with varying temporal overlap (i.e., different SOAs) and different task versions are combined in different task pairs, indicated by task-version cues. The authors used two cues per task pair and found typical dual-task interference, indicating that dual-task performance is impaired as a function of increased temporal overlap. Furthermore, they observed cue switch costs, possibly reflecting perceptual cue priming. Importantly, however, there were also task-pair switch costs that occurred even when controlling for cue switching. This suggests that task-pair switching per se produces a performance cost that cannot be reduced to the costs of cue switching (Logan & Schneider, 2006). Additionally, the authors employed a go/no-go-like manipulation and observed task-pair switch costs even after no-go trials where subjects prepared for a task pair but did not perform this pair. Thus, there is evidence showing that task pairs are already activated before performing a dual task. Hirsch et al.’s data provided evidence for a representation, including the task-pair set, that is organized at a hierarchically different level than the processes of the individual component tasks of a dual task. This conclusion confirms previous studies by this group of authors (Hirsch et al., 2017, 2018). In sum, this conclusion of task-pair representations on a different level than the representations of the individual component tasks is generally consistent with the assumption of task-order representations. This is also consistent with the assumption of the DTCA framework that DTC processes such as task-pair representations (Level b) are separable from the component tasks, constituting a dual task (Level a).

Further empirical evidence for higher-order representations, including task-order information, on the DTC-Level b stems from a study by Kübler et al. (2022a). In this study, we applied a dual task with randomly variable task orders consisting of an auditory and a visual task. In addition to task order, the visual task varied randomly between two task versions from trial to trial, while the auditory task remained constant in a first experiment. In a second experiment, the auditory task varied between two auditory task versions, and the visual task was constant, while both component tasks varied in a third experiment. In all experiments, performance benefits occurred in trials with a repeated task order relative to trials with a switched task order, irrespective of the repeated or the switched component task version. This indicates that task-order representations in dual tasks contain task-order information but no exclusive information about the individual component tasks.

We also demonstrated that these task-order representations require resources in the working memory (Kübler et al., 2022b). In detail, theories on task-order coordination suggest that these DTC processes rely on order representations that are actively maintained and processed in this memory component. Importantly, as working memory has only limited capacity (Baddeley, 2003; Cowan, 2010), this explanation conceptualizes task-order coordination as a resource-dependent process. Kübler et al. (2022b) empirically demonstrated this contribution of working memory as follows: In their study, the load on working memory resources was varied either by tasks with different numbers of stimulus–response mappings or by a working memory task in addition to the dual task (i.e., maintaining a number sequence in working memory during performing dual-task blocks). The results of both manipulations demonstrated that the performance benefits for trials with repeated task orders relative to trials with switched task orders were reduced under high compared with low working memory load. That is, the benefit of repeating task orders is reduced if the previous task-order set cannot be easily maintained because of the high working memory load. This reduction results from the fact that task-order representations of the previous trials cannot be applied under repeated task-order conditions when a high amount of information occupies the working memory. These results were elegantly confirmed in a reverse relationship by Otermans et al. (2022). The authors showed that the working memory span was lower under conditions of dual tasks with random task orders in comparison to dual tasks with constant task orders (Experiment 2). Importantly, comparing different conditions of random order dual tasks, this span was also lower when the number of order switches was high versus when the number of order switches was low (Experiment 3).

The results of the previous section confirm the assumption that task-order information for processing DTC relies on working memory resources. These results also complete the review of literature about (1) dual-task practice and (2) dual-task processing without practice components in this section, pointing out the existence of DTC processes (Level b). This section also specified the characteristics of these processes and their separation from the processing streams of the component tasks (Level a), which is a key assumption of the DTCA framework.

From a broader perspective beyond the DTCA framework and the overlapping task paradigm, this key component is explicitly consistent with previous cognitive architectures, such as EPIC (Meyer & Kieras, 1997), ECTVA (Logan & Gordon, 2001), ACT-R (Byrne & Anderson, 2001), and threaded cognition (Salvucci & Taatgen, 2008). In EPIC, DTC is explained through an independent mechanism known as the strategic response deferment model. This response deferment strategy is assumed to be supervised by an executive process that controls the selection of component task responses (Meyer & Kieras, 1997). Importantly, EPIC enables the possibility that the executive process may interact with working memory load, as components of the task are stored in working memory until released for a response. Unlike the DTCA framework, EPIC establishes an explicit and formal connection between working memory load, DTC, and the differentiation between mechanisms underlying component tasks. Further, EPIC does not exclude the possibility of full parallel processing of component tasks. Similarly, the ECTVA framework of Logan and Gordan explains how subordinate processes in component tasks are programmed by a set of parameters and how executive processing (equivalent to DTC) programs these parameters. This way, ECTVA allows serial as well as parallel component task processing, depending on the parameter programming. In contrast, ACT-R (Byrne & Anderson, 2001) assumes a mechanism similar to DTC, with the crucial addition that production rules must be carried out serially, resembling a central bottleneck such as in the DTCA framework. Notably, recent neural network models have also presented advancements in DTC, wherein the limitations in the capability to coordinate multiple tasks can be explained by representation sharing between tasks. Neural systems trade the benefits of shared representation for rapid learning and generalization against constraints on performance multiple tasks (Musslick & Cohen, 2021).

Similar to EPIC, the threaded cognition model (Salvucci & Taatgen, 2008) assumes the independence of component processes and DTC. In this architecture, DTC is carried out by architectural rules without the involvement of a central executive or the assumption of a central bottleneck. The threaded cognition model proposes that task components are scheduled in a greedy and polite manner to occupy resources. Therefore, the model suggests that DTC occurs independently, although it allows for interactions between changes in the component processes and the scheduling scheme. Taatgen and Lee (2003) explain the observed dual-task practice effects, which are surveyed in the present review, in terms of production compilation, which involves chunking the processes for component tasks. Importantly, contrary to the implications in the current paper, the mechanism of production compilation suggests that improvements in multitasking cannot be solely explained by enhancements in the component tasks. Instead, the chunking of task components reduces the need to schedule retrievals from declarative memory, enabling more flexible scheduling strategies. Thus, the architectures EPIC, ECTVA, ACT-R, and threated cognition can provide a high level of predictive precision that goes even beyond the current DTCA framework. The latter rather aims at structuring different processing levels for dual tasks (i.e., Levels a, b, and c).

## Chara﻿cteristics of coordination adjustment

After demonstrating the separability of a set of DTC processes from the set of processes within the component tasks, the DTCA framework assumes that there is a level that is responsible for adjusting these coordination processes. Figure 1 illustrates this Level c of DTCA. In this section, I first present empirical results that demonstrate this adjustment in the overlapping task context to illustrate how coordination adjustment is manifested in this context. Secondly, I outline how such adjustment is realized in contexts outside dual tasks (e.g., in conflict paradigms and paradigms assessing cognitive control). Thirdly, a literature review aims at illustrating the similarities and differences between DTC and DTCA, opening up the question of whether it is warranted to add an additional meta-control adjustment Level c (in addition to DTC on Level b) to the DTCA framework.

### ﻿Empirical findings illustrating coordination adjustment

One of the first empirical illustrations of DTCA was illustrated as follows: To adjust DTC, we implemented a dual task with a combination of a visual number task and an auditory tone task. This dual-task combination was presented with a random stimulus order under two instruction conditions: Participants were told to respond either in the order of stimulus presentation or in the order they preferred (Strobach et al., 2018). RTs generally decreased under the preferred condition in comparison to the condition with responses according to the stimulus-order presentation, in particular under long SOA conditions. Under the same conditions, participants produced more response reversals (i.e., responses carried out in the opposite order as the stimulus order would mandate). These findings might indicate that meta-control of DTCA is engaged in determining task order when the participants are explicitly told to respond in the order of stimulus presentation (in comparison to when they perform a preferred order) and when an increased DTC resource is required. This determination is assumed to be a coordination adjustment since it modulates the coordination of dual tasks.

**Fig. 3:**
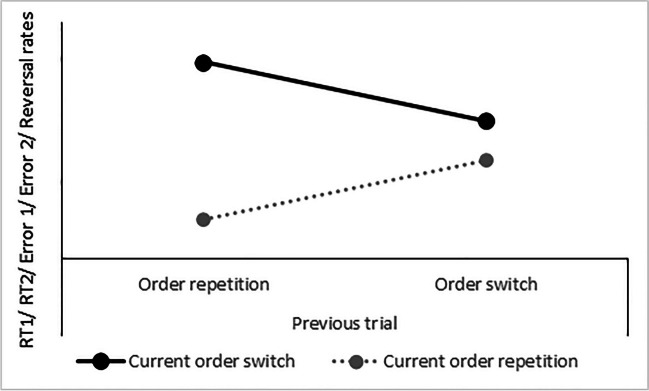
Illustration of hypothetical data showing the switch adjustment effect. *Note.* The order switch costs (i.e., the difference between current repetition-order trials and current switch-order trials) are reduced after previous switch-order trials in comparison to previous repetition-order trials. The switch adjustment effect is illustrated for reaction times on a first task (RT1), reaction times on a second task (RT2), error rates on a first task (Error 1), error rates on a second task (Error 2), and reversal rates. Note that the switch adjustment effect comes with different generic forms for different data

**Fig. 4:**
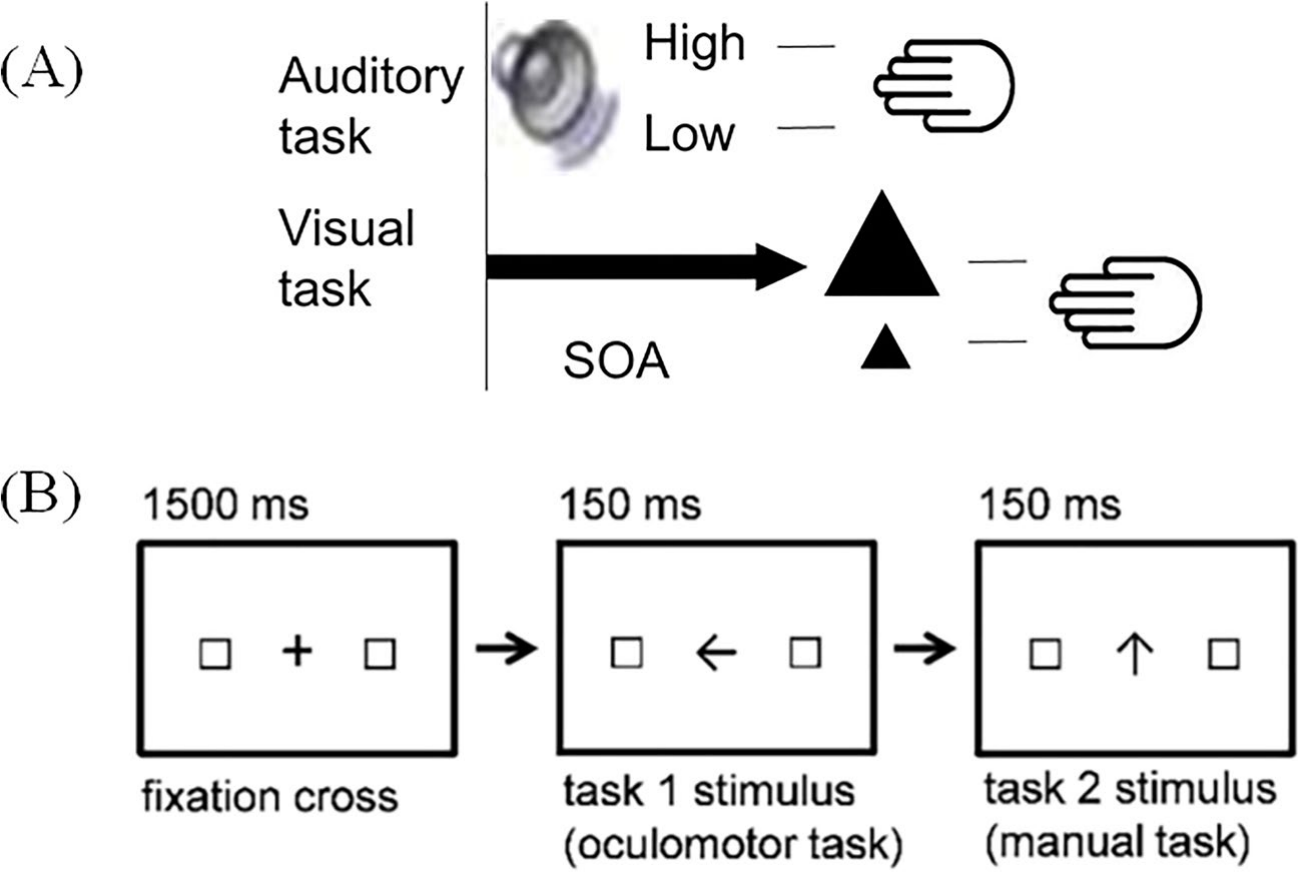
Illustration of component-task combinations in which the switch adjustment effect was demonstrated. *Note.* Panel (**A**): Visual and auditory tasks with manual-manual response combinations in Strobach et al. (2021) and Strobach and Wendt (2022). Panel (**B**): Tasks with oculomotor and manual response combinations in Strobach et al. (2023). The tasks combinations in **A** and **B** are also presented in alternative orders

Another very recent approach to illustrate the adjustment of DTC experimentally is the fact that order switch costs between switch-order versus repetition-order conditions in a current trial are modulated by the order sequence status of the predecessor trial. Specifically, Strobach and colleagues (Strobach et al., 2021, 2023; Strobach & Wendt, 2022) demonstrated reduced order switch costs in current trial N when the previous trial *N* − 1 itself involved a switch of task order (i.e., the task order changed from the penultimate trial *N* − 2 to previous trial *N* − 1) compared with when this previous trial *N* − 1 involved a repetition (i.e., the task order did not change from the penultimate trial *N* − 2 to previous trial *N* − 1). This *switch adjustment effect* is illustrated in Fig. 3, and the component-task combinations in which this effect was demonstrated are depicted in Fig. 4. The switch adjustment effect occurred for

- both component tasks in dual-task situations (i.e., Task 1 and Task 2);
- RTs, error rates, and response reversal rates on manual responses (Strobach et al., 2021, 2023; Strobach & Wendt, 2022), as well as on oculomotor responses (Strobach et al., 2023); and
- situations with component tasks that were not differentiated according to dominance (Strobach et al., 2021; Strobach & Wendt, 2022) as well as in situations with component tasks of different dominances (Strobach et al., 2023).

Additionally, the switch adjustment effect occurred for several experimental conditions that this review elaborates more on below. That is, in the overlapping task context, this effect

- occurred for short and relatively long intervals between task stimuli (i.e., different SOAs; Strobach et al., 2021);
- was observed for two-choice tasks and three-choice tasks (i.e., different memory loads; Strobach et al., 2021);
- was observed for short or long intervals between trials (i.e., different intertrial intervals; Strobach & Wendt, 2022); and
- was evident in situations with task orders of equal preference (Strobach et al., 2021; Strobach & Wendt, 2022) as well as in situations with task orders of nonequal preferences (i.e., different task-order preferences; Strobach et al., 2023).

Alternative pivotal contexts for the illustration of DTCA might be the free concurrent dual-task paradigm (Brüning et al., 2020) and the paradigm of dual-memory retrieval (Orscheschek et al., 2019; Strobach et al., 2014b). Research utilizing these paradigms has demonstrated that participants tend to organize their voluntary dual-task behavior through strategies such as blocking, switching, or response grouping (Brüning et al., 2021; Brüning & Manzey, 2018), as well as response grouping and nongrouping (Nino & Rickard, 2003), respectively.

### ﻿Cognitive control adjustment outside dual tasks

The switch adjustment effect of reduced order switch costs after task-order switches versus after task-order repetitions (Strobach et al., 2021) basically resembles the Gratton effect phenomenon in investigations of trial-wise cognitive control adjustment in conflict paradigms (Gratton et al., 1992) and is based on the assumption of domain-general control adaptation theories (Botvinick et al., 2001; Miyake et al., 2000). These conflict paradigms have demonstrated ample evidence for cognitive processing of stimulus aspects that are irrelevant to a current task (i.e., aspects that contain information not necessary for the currently correct task performance). Prominent examples of this processing of irrelevant information can be seen in relative performance impairments when a distractor stimulus feature, such as a word in the Stroop task (Stroop, 1935), a stimulus object adjacent to the target stimulus in the Eriksen flanker task (Eriksen & Eriksen, 1974), or the stimulus location in the Simon task (Simon & Small, 1969), is associated with an incorrect response in comparison with conditions without features of incorrect response information. For instance, when participants have to respond to the ink of Stroop color words, performance is impaired under incongruent conditions (i.e., ink and word meaning do not match, such as in RED) in comparison to congruent conditions (i.e., ink and word meaning match, such as in BLACK), generally resulting in a congruency effect in the form of the prominent Stroop effect in this task. Such result patterns suggest distractor-related response activation that interferes with responding to the target stimulus feature.

It has been shown that the size of the congruency effects depends, among others, on the recency of congruent and incongruent trials in conflict paradigms. More precisely, congruency effects are reduced in a current trial *N* after experiencing an incongruent stimulus in a previous trial *N* – 1 in comparison with these effects after a congruent stimulus in a previous trial (i.e., the Gratton effect; Gratton et al., 1992; Mayr & Awh, 2009). Since congruency effects are smaller after incongruent trials, researchers infer that adaptive processes are putatively triggered and there is greater recruitment of cognitive control; this greater recruitment is the consequence of adjustment of executive control (Chiu & Egner, 2019). Such short-lived, dynamic adjustment of executive control are particularly important for matching processing modes (e.g., an increased focus on ink information than on word meaning information in color Stroop stimuli) as a consequence of changing environmental demands and/or as a response to conflict experience as well as performance monitoring signals (e.g., a conflict; Botvinick et al., 2001; Goschke, 2003).

Research on executive control adjustment is concerned with how control is regulated in a dynamic and time-varying manner to capture both the need to deal with a changing environment as well as the notion that executive control is demanding and costly and should be imposed only as much as necessary (Shenhav et al., 2013). The seminal conflict-monitoring account (Botvinick et al., 2001) proposed that conflict between task information is detected by a top-down conflict monitor, which signals the need for adaptation to specific executive control modules. While other theoretical accounts discuss the need for the existence of such modules and the integration between control modules and task-specific learning (e.g., contingency learning, associative learning, episodic memory of stimulus–response episodes; for a recent discussion, see Braem et al., 2019), most control theories assume some sort of adjustment of control to just-experienced conflict between competing information to explain the Gratton effect. These control adjustment processes of the conflict-monitoring account (Botvinick et al., 2001) are usually assumed to be rather generic in nature, so it seems plausible to assume that the adjustment in DTC is coordinated or initiated by the very same processes as the adjustment in conflict processing. Thus, it might be that similar dynamic adjustment in cognitive control regulate conflict processing and DTC. In particular, this DTCA might regulate task-order coordination across trials (e.g., Strobach et al., 2021). This adjustment would explain modulations in the order switch costs, as illustrated by the switch adjustment effect.

Given that transfer of practice effects is a major issue in the cognitive training literature and the literature on DTC, it is relevant to analyze the existing literature on DTCA with a focus on transfer. According to the author’s knowledge, there is no study investigating practice effects on the switch adjustment effect. However, there are informative studies in the literature beyond dual tasks. First, in task-switching studies (Kiesel et al., 2010; Koch et al., 2018), there is research on the so-called switch-readiness based on manipulations of the proportion of task switches within an experimental block. With frequent task switches in comparison to infrequent task switches, there is a reduction in task-switch costs (i.e., longer RTs in task-switch trials in comparison to RTs in task-repetition trials). Importantly, recent studies showed that this “coordination bias” does not transfer to contexts with new component tasks (Chiu & Egner, 2019). This seems to suggest that this type of meta-control is specific to the situation at hand and does not transfer easily.

In contrast, the response execution mode in dual-retrieval practice situations (Orscheschek et al., 2019; Strobach et al., 2014b) is either some kind of chunked retrieval of two responses from long-term memory with a grouped and synchronized response execution (grouping mode) or to execute each response sequentially as soon as it is retrieved from long-term memory (non-grouping mode). Studies showed that the non-grouping and grouping response modes can be transferred after dual-retrieval practice to different sets of cues. So, these meta-control modes during dual-retrieval practice are transferable to some extent. Thus, the studies elaborated in this section allow for different conclusions regarding the transfer of meta-control skills. Furthermore, it would be relevant to adapt the current assumptions about practice on DTCA to early-perceptual phenomena of visual dominance effects in the context of visual and auditory targets (Colavita, 1974; Sinnett et al., 2007). However, discussing these studies in a wider context might make the present study even more interesting for a more general readership.

### Similarities and differences between dual-task coordination and coordination adjustment

Before there is research on the specific nature of the mechanisms of the switch adjustment effect, it is relevant to justify the existence of processes that are associated with DTCA. That is, it is relevant to show that processes of DTCA on Level c are separable from DTC processes on Level b of the DTCA framework. Therefore, I ask whether there are empirical findings that not only illustrate DTCA, but whether there is evidence that justifies the separability of DTC processes and cognitive meta-control that adjusts those coordination processes.

Focusing on practice and nonpractice studies, the efficient task instantiation model (Schubert & Strobach, 2018) assumes that the practice-related optimization of DTC skills mainly improves Task 2 performance in a dual-task situation, while DTCA showed effects on Task 1 as well as Task 2 in nonpractice studies (e.g., Strobach et al., 2021). Thus, this set of empirical findings indirectly shows differences between DTC and DTCA findings, which is consistent with the assumed separability of these sets of processes. Furthermore, there are empirical indicators that support this separability in nonpractice studies exclusively. These indicators are derived from four experimental factors—namely, (1) SOA, (2) memory load, (3) intertrial interval, and (4) task-order preference—and their impact on DTC processes and DTCA processes. An impact indicates that these experimental factors modulate these sets of processes, whereas a lack of impact is supported by the finding of no modulation of these sets due to these factors. The finding of different impacts of experimental factors on both sets of processes is consistent with the assumption of different characteristics and thus distinct mechanisms underlying these sets. For example, an experimental factor has an effect on DTC but not on DTCA. This dissociation could point to distinct mechanisms for these sets of processes, which is consistent with the assumption of separable sets. This separation is consistent with the DTCA framework, which distinguishes between DTC on Level b and DTCA on Level c.

First, Luria and Meiran (2003, 2006), as well as Strobach et al. (2021), demonstrated that the PRP effect in the second task of PRP situations is reduced under conditions of task order repetitions in comparison to task order switches. So, when the current trial *N* and the previous trial *N* – 1 represent the same task order compared with different task orders across trials, PRP effects are reduced. This reduction shows that DTC processes affect the effect of SOA on performance in Task 2. However, this reduction of the PRP effect based on the task order in the current trial in relation to the previous trial is not further modulated by the task order condition of the previous trials in relation to the penultimate trial (i.e., there is no effect of the “order sequence” status of the predecessor trial on the modulation of the PRP effect; Strobach et al., 2021). This set of findings demonstrates distinct characteristics of DTC processes (i.e., the current task-order condition affects the PRP effect) and the adjustment of those processes (i.e., the interaction of previous and current task-order conditions does not affect the PRP effect). Thus, there is no evidence from the SOA factor that DTC processes and meta-control that adjusts those coordination processes underlie the same characteristics and mechanisms. Rather, these findings support the assumption that the coordination processes and their adjustment represent different sets of mechanisms, which is consistent with key predictions of the DTCA framework of separable DTC and DTCA.

Second, we have demonstrated that task-order information is actively maintained and processed in task-order representations that require resources from the working memory (Kübler et al., 2022b). We empirically demonstrated this contribution of working memory by varying the task difficulty and, in particular, the memory load of the component tasks by varying the number of stimulus–response task mappings (i.e., high versus low mapping numbers refer to high versus low memory load, respectively). The performance benefits for trials with repeated task orders relative to trials with switched task orders were reduced under high compared with low working memory loads (i.e., order switch costs were reduced). Thus, memory load has an impact on DTC. Importantly, Strobach et al. (2021) provided no evidence that the interaction of task order in the previous trial *N* − 1 and in the current trial *N* was modulated by different stimulus–response mapping numbers. Thus, there is no evidence of an impact of memory load on DTCA. The latter is problematic when assuming that cognitive control is involved in DTCA since cognitive control, at least in the context of conflict monitoring, would hypothesize relying on working memory as a mediating representation of task goals (Botvinick et al., 2001). However, the latter study of Strobach et al. did not provide evidence of an impact of the memory load variation on order switch costs in the current trial *N* and thus DTC (in contrast to the findings of Kübler et al., 2022b). So, based on this inconsistent set of findings, it remains an open issue at the moment, whether DTC processes and their adjustment represent distinct sets of mechanisms.

Third, the combination of intertrial interval, current trial order, and previous trial order was not significant in Strobach and Wendt (2022). Thus, the switch adjustment effect is not modulated by the time intervals between trials (i.e., these intervals do not affect DTCA). In contrast, the combination of these intervals and the current trial order demonstrated a trend for decreased order switch costs after shorter intertrial intervals in comparison with longer intertrial intervals. Thus, the modulation of the interval between tasks demonstrated a trend for a modulation of DTC. So, there are first indicators for distinct mechanisms of the impact of the intertrial interval on DTC (Level b) and DTCA (Level c) in the DTCA framework.

Fourth, it has been shown that task-order scheduling processes take task-specific characteristics into account: task order switches were easier and order switch costs were lower when switching to a preferred (versus nonpreferred) task order (Huestegge et al., 2021). In addition, Strobach et al. (2023) asked whether a task order switch in a previous trial facilitates a task order switch in a current trial and takes task-specific and task-order-specific characteristics into account. Based on three experiments involving task order switches between a preferred (dominant oculomotor task prior to a nondominant manual/pedal task) and a nonpreferred (vice versa) order, the authors replicated the finding that task order switching (in Trial *N*) is facilitated after a previous switch (versus repetition in Trial *N* − 1) in task order. However, there was no substantial evidence in favor of a significant difference when switching to the preferred versus nonpreferred order and in the analyses of the dominant oculomotor task and the nondominant manual task. This indicates different characteristics underlying the control of immediate task order configuration (indexed by order switch costs) and the sequential modulation of these costs based on the task order sequence in the previous trial. In essence, these findings support the assumption that the coordination processes and their adjustment represent distinct sets of mechanisms. According to the applied logic, these findings are consistent with key predictions of the DTCA framework and a separation of Level b (DTC) and Level c (DTCA).

Note that several arguments in the present section are of the nature that an experimental manipulation (e.g., the manipulation of the intertrial interval in Strobach & Wendt, 2022) affects DTC, but not DTCA. It seems that mostly effects for the more low-level DTC are shown, while effects for the more high-level DTCA are absent and generally DTCA does not seem to “respond” to any variation so far and is simply a robust but invariable finding. This imbalance is a bit problematic because one would expect somewhat smaller effects for the more high-level DTCA, and the arguments are based on null effects. It would be very strong evidence if there is a manipulation that affects DTCA but not DTC. Since the present manuscript does not include new data, lacking systematic data synthesis such as systematic meta-analyses do not offer insights into the validity of the applied logic in the present sections. Nevertheless, this lack of insight does not affect the conclusions about the existence of the switch adjustment effect that are based on statistically significant results.

Summarizing the present section, there are empirical demonstrations of the phenomenon of DTCA (Strobach et al., 2018, 2021, 2023; Strobach & Wendt, 2022). These demonstrations reflect consistent demonstrations in contexts beyond dual tasks—namely, conflict paradigms (e.g., Botvinick et al., 2001; Gratton et al., 1992; Kerns et al., 2004). Thus, these phenomena in dual tasks and beyond might be associated with the same underlying mechanisms. However, I illustrated some factors in dual tasks that point to distinct mechanisms underlying DTC processes and meta-control processes of DTCA. This difference is consistent with the assumptions of the DTCA framework, separating the Levels b and c. However, not all experimental factors in empirical studies (e.g., memory load) provide conclusive evidence for such a distinction. So, additional empirical tests are required to allow conclusive assumptions about the DTCA framework and its validity.

One first step to test the validity of the DTCA framework could be to investigate the separability of processes within the component tasks versus DTC processes/processes of DTCA. With reference to paradigms of adaptive cognitive control, numerous researchers have pointed out that classic asserted indices of adaptive control in conflict tasks can often be interpreted by the characteristics of the constituent component tasks in terms of more basic stimulus–stimulus or stimulus–response learning processes (for a review, see Schmidt, 2019). These considerations have led to various theoretical discussions as to how such forms of lower-level task-set learning relate to cognitive control and might argue against such an independent control component. However, studies in this domain generally agree that manipulations that promote learning at this task-set level are relatively easy to avoid. Therefore, if researchers want to study adaptive control independent of low-level learning in the context of DTC (Braem et al., 2019), one recommendation is that they employ paradigms that are designed to minimize opportunities for exploiting stimulus–response or stimulus–stimulus associations (e.g., by avoiding stimulus–response or stimulus–stimulus repetitions across trials).

Another key assumption of the DTCA framework is the existence of processes of DTCA that (meta-)control a separate set of DTC processes. The basic idea for investigating this assumption is the following: If these sets of processes are separate from each other, then they could have distinct mechanisms, and they should thus respond differently to the same experimental manipulations. For instance, future studies could investigate how similar or dissimilar DTC and DTCA is related to working memory resources. This endeavor can be realized with two experimental approaches: How do DTC and DTCA react to (1) dual tasks with a variation of the number of stimulus–response mappings within the component tasks (e.g., four- versus two-forced choice tasks) and (2) dual tasks with variation due to an additional working memory task? The investigation of practice could be another approach to disentangle DTC and DTCA. This approach starts with the conclusion that DTC can generally be improved with practice (Strobach, 2020). However, according to the separability assumption, practice should have no impact on coordination adjustment. Findings from attentional adjustments during task switching showed no evidence for practice effects (while task switching itself did; Strobach et al., 2020), which would be consistent with this assumption and would show immunity of adjustment processes to practice.

## ﻿Summary

The current paper presents the DTCA framework. In this framework, there are active DTC processes (Level b) that work on the scheduling of capacity-limited processing stages of two simultaneous tasks in the overlapping task paradigm (Level a). However, there are recent findings that point to a meta-cognitive control level in addition to these active coordination processes (Level c). This additional level’s responsibility is to adjust DTC to efficiently schedule capacity-limited stages. I provide reviews of evidence focusing on the existence of DTC processes and processes of DTCA. The remainder of the paper elaborates on preliminary findings, which point to a separability of these sets of processes, which is a key assumption of the DTCA framework, and to a meta-control level of DTC.

## Data Availability

Not applicable.

## References

[CR1] Ahissar, M., Laiwand, R., & Hochstein, S. (2001). Attentional demands following perceptual skill training. *Psychological Science, 12*(1), 56–62. 11294229 10.1111/1467-9280.00310

[CR2] Allen, R. J. (2022). Short-term and working memory. In S. D. Sala (Ed.), *Encyclopedia of behavioral neuroscience* (2nd ed., pp. 470–478). Elsevier.

[CR3] Anderson, J. R., Taatgen, N. A., & Byrne, M. D. (2005). Learning to achieve perfect timesharing: Architectural implications of Hazeltine, Teague, and Ivry (2002). *Journal of Experimental Psychology: Human Perception and Performance, 31*(4), 749–761. 16131247 10.1037/0096-1523.31.4.749

[CR4] Baddeley, A. (2003). Working memory: Looking back and looking forward. *Nature Reviews Neuroscience, 4*(10), 829–839. 14523382 10.1038/nrn1201

[CR5] Botvinick, M. M., Braver, T. S., Barch, D. M., Carter, C. S., & Cohen, J. D. (2001). Conflict monitoring and cognitive control. *Psychological Review, 108*(3), 624–652. 11488380 10.1037/0033-295x.108.3.624

[CR6] Braem, S., Bugg, J. M., Schmidt, J. R., Crump, M. J., Weissman, D. H., Notebaert, W., & Egner, T. (2019). Measuring adaptive control in conflict tasks. *Trends in Cognitive Sciences, 23*(9), 769–783. 31331794 10.1016/j.tics.2019.07.002 PMC6699878

[CR7] Bratzke, D., Rolke, B., & Ulrich, R. (2009). The source of execution-related dual-task interference: Motor bottleneck or response monitoring? *Journal of Experimental Psychology: Human Perception and Performance, 35*(5), 1413–1426. 19803646 10.1037/a0015874

[CR8] Brüning, J., & Manzey, D. (2018). Flexibility of individual multitasking strategies in task-switching with preview: Are preferences for serial versus overlapping task processing dependent on between-task conflict? *Psychological Research, 82*(1), 92–108. 28983726 10.1007/s00426-017-0924-0

[CR9] Brüning, J., Mückstein, M., & Manzey, D. (2020). Multitasking strategies make the difference: Separating processing-code resources boosts multitasking efficiency when individuals prefer to interleave tasks in free concurrent dual tasking. *Journal of Experimental Psychology: Human Perception and Performance, 46*(12), 1411–1433. 10.1037/xhp0000865 33090838

[CR10] Brüning, J., Reissland, J., & Manzey, D. (2021). Individual preferences for task coordination strategies in multitasking: Exploring the link between preferred modes of processing and strategies of response organization. *Psychological Research, 85*, 577–591. 32006093 10.1007/s00426-020-01291-7 PMC7900073

[CR11] Byrne, M. D., & Anderson, J. R. (2001). Serial modules in parallel: The psychological refractory period and perfect time-sharing. *Psychological Review, 108*(4), 847–869. 11699122 10.1037/0033-295x.108.4.847

[CR12] Chiu, Y.-C., & Egner, T. (2019). Cortical and subcortical contributions to context-control learning. *Neuroscience & Biobehavioral Reviews, 99*, 33–41. 30685484 10.1016/j.neubiorev.2019.01.019 PMC6399056

[CR13] Colavita, F. B. (1974). Human sensory dominance. *Perception & Psychophysics, 16*(2), 409–412.

[CR14] Cowan, N. (2001). Metatheory of storage capacity limits. *Behavioral and Brain Sciences, 24*(01), 154–176. 10.1017/s0140525x01003922 11515286

[CR15] Cowan, N. (2010). The magical mystery four: How is working memory capacity limited, and why? *Current Directions in Psychological Science, 19*(1), 51–57. 20445769 10.1177/0963721409359277 PMC2864034

[CR16] Cowan, N., Elliott, E. M., Saults, J. S., Morey, C. C., Mattox, S., Hismjatullina, A., & Conway, A. R. (2005). On the capacity of attention: Its estimation and its role in working memory and cognitive aptitudes. *Cognitive Psychology, 51*(1), 42–100. 16039935 10.1016/j.cogpsych.2004.12.001 PMC2673732

[CR17] de Jong, R. (1993). Multiple bottlenecks in overlapping task performance. *Journal of Experimental Psychology: Human Perception and Performance, 19*(5), 965–980. 8228846 10.1037//0096-1523.19.5.965

[CR18] de Jong, R. (1995). The role of preparation in overlapping-task performance. *The Quarterly Journal of Experimental Psychology A: Human Experimental Psychology, 48A*(1), 2–25. 10.1080/14640749508401372 7754082

[CR19] de Jong, R. (2000). An intention-activation account of residual switch costs. In S. Monsell & J. Driver (Eds.), *Control of cognitive processes: Attention and performance XVIII* (pp. 357–376). MIT Press.

[CR20] Dux, P. E., Tombu, M. N., Harrison, S., Rogers, B. P., Tong, F., & Marois, R. (2009). Training improves multitasking performance by increasing the speed of information processing in human prefrontal cortex. *Neuron, 63*, 127–138. 19607798 10.1016/j.neuron.2009.06.005 PMC2713348

[CR21] Eriksen, & Eriksen, C. W. (1974). Effects of noise letters upon the identification of a target letter in a nonsearch task. *Perception & Psychophysics, 16*(1), 143–149.

[CR22] Frings, C., Hommel, B., Koch, I., Rothermund, K., Dignath, D., Giesen, C., & Philipp, A. M. (2020). Binding and retrieval in action control (BRAC). *Trends in Cognitive Sciences, 24*(5), 375–387. 32298623 10.1016/j.tics.2020.02.004

[CR23] Garner, K., Tombu, M., & Dux, P. (2014). The influence of training on the attentional blink and psychological refractory period. *Attention, Perception, & Psychophysics, 76*, 979–999. 10.3758/s13414-014-0638-y 24627208

[CR24] Goschke, T. (2003). Voluntary action and cognitive control from a cognitive neuroscience perspective. In S. Maasen, W. Prinz, & G. Roth (Eds.), *Voluntary action: Brains, minds and sociality* (pp. 49–85). Oxford University Press.

[CR25] Gratton, G., Coles, M. G., & Donchin, E. (1992). Optimizing the use of information: Strategic control of activation of responses. *Journal of Experimental Psychology: General, 121*(4), 480–506. 1431740 10.1037//0096-3445.121.4.480

[CR26] Hartley, A. A., Maquestiaux, F., & Butts, N. S. (2011). A demonstration of dual-task performance without interference in some older adults. *Psychology and Aging, 26*(1), 181–187. 21299304 10.1037/a0021497

[CR27] Hazeltine, E., Ruthruff, E., & Remington, R. W. (2006). The role of input and output modality pairings in dual-task performance: Evidence for content-dependent central interference. *Cognitive Psychology, 52*(4), 291–345. 16581054 10.1016/j.cogpsych.2005.11.001

[CR28] Hendrich, E., Strobach, T., Buss, M., Müller, H. J., & Schubert, T. (2012). Temporal-order judgment of visual and auditory stimuli: Modulations in situations with and without stimulus discrimination. *Frontiers in Integrative Neuroscience, 6*, 63. 22936902 10.3389/fnint.2012.00063 PMC3427541

[CR29] Hirsch, P., Nolden, S., & Koch, I. (2017). Higher-order cognitive control in dual tasks: Evidence from task-pair switching. *Journal of Experimental Psychology: Human Perception and Performance, 43*(3), 569–580. 28080113 10.1037/xhp0000309

[CR30] Hirsch, P., Nolden, S., Philipp, A. M., & Koch, I. (2018). Hierarchical task organization in dual tasks: Evidence for higher level task representations. *Psychological Research, 82*(4), 759–770. 28285364 10.1007/s00426-017-0851-0

[CR31] Hirsch, P., Roesch, C., & Koch, I. (2021). Evidence for a multicomponent hierarchical representation of dual tasks. *Memory & Cognition, 49*, 350–363. 32989661 10.3758/s13421-020-01097-3 PMC7886772

[CR32] Hirst, W., Spelke, E. S., Reaves, C. C., Caharack, G., & Neisser, U. (1980). Dividing attention without alternation or automaticity. *Journal of Experimental Psychology: General, 109*(1), 98–117.

[CR33] Hommel, B. (2004). Event files: Feature binding in and across perception and action. *Trends in Cognitive Sciences, 8*(11), 494–500. 15491903 10.1016/j.tics.2004.08.007

[CR34] Huestegge, L., Hoffmann, M. A., & Strobach, T. (2021). Task-order representations in dual tasks: Separate or integrated with component task sets? *Quarterly Journal of Experimental Psychology, 74*(12), 2097–2111. 10.1177/17470218211018416 34024211

[CR35] Huestegge, L., & Koch, I. (2010). Crossmodal action selection: Evidence from dual-task compatibility. *Memory & Cognition, 38*(4), 493–501. 20516229 10.3758/MC.38.4.493

[CR36] Kamienkowski, J. E., Pashler, H., Dehaene, S., & Sigman, M. (2011). Effects of practice on task architecture: Combined evidence from interference experiments and random-walk models of decision making. *Cognition, 119*(1), 81–95. 21295773 10.1016/j.cognition.2010.12.010

[CR37] Kerns, J. G., Cohen, J. D., MacDonald, A. W., III, Cho, R. Y., Stenger, V. A., & Carter, C. S. (2004). Anterior cingulate conflict monitoring and adjustments in control. *Science, 303*(5660), 1023–1026. 14963333 10.1126/science.1089910

[CR38] Kiesel, A., Steinhauser, M., Wendt, M., Falkenstein, M., Jost, K., Philipp, A. M., & Koch, I. (2010). Control and interference in task switching—A review. *Psychological Bulletin, 136*(5), 849–874. 20804238 10.1037/a0019842

[CR39] Koch, I., Poljac, E., Müller, H., & Kiesel, A. (2018). Cognitive structure, flexibility, and plasticity in human multitasking—An integrative review of dual-task and task-switching research. *Psychological Bulletin,**144*(6), 557–583. 10.1037/bul0000144 29517261 10.1037/bul0000144

[CR40] Kramer, A. F., Larish, J. F., & Strayer, D. L. (1995). Training for attentional control in dual task settings: A comparison of young and old adults. *Journal of Experimental Psychology: Applied,**1*(1), 50–76. 10.1037/1076-898X.1.1.50 10.1037/1076-898X.1.1.50

[CR41] Kübler, S., Reimer, C. B., Strobach, T., & Schubert, T. (2018). The impact of free-order and sequential-order instructions on task-order regulation in dual tasks. *Psychological Research, 82*(1), 40–53. 28856434 10.1007/s00426-017-0910-6

[CR42] Kübler, S., Strobach, T., & Schubert, T. (2022a). On the organization of task-order and task-specific information in dual-task situations. *Journal of Experimental Psychology: Human Perception and Performance, 48*(1), 94–113 10.1037/xhp0000969 35073146 10.1037/xhp0000969

[CR43] Kübler, S., Strobach, T., & Schubert, T. (2022b). The role of working memory for task-order coordination in dual-task situations. *Psychological Research,**86*(2), 452–473. 10.1007/s00426-021-01517-2 33884485 10.1007/s00426-021-01517-2 PMC8885531

[CR44] Leonhard, T., Fernández, S. R., Ulrich, R., & Miller, J. (2011). Dual-task processing when task 1 is hard and task 2 is easy: Reversed central processing order? *Journal of Experimental Psychology: Human Perception and Performance, 37*(1), 115–136. 20718575 10.1037/a0019238

[CR45] Liepelt, R., Strobach, T., Frensch, P., & Schubert, T. (2011). Improved intertask coordination after extensive dual-task practice. *The Quarterly Journal of Experimental Psychology,**64*(7), 1251–1272. 10.1080/17470218.2010.543284 21462091 10.1080/17470218.2010.543284

[CR46] Logan, G. D., & Bundesen, C. (2003). Clever homunculus: Is there an endogenous act of control in the explicit task-cuing procedure? *Journal of Experimental Psychology: Human Perception and Performance, 29*(3), 575–599. 12848327 10.1037/0096-1523.29.3.575

[CR47] Logan, G. D., & Gordon, R. D. (2001). Executive control of visual attention in dual-task situations. *Psychological Review,**108*(2), 393–434. 10.1037/0033-295X.108.2.393 11381835 10.1037/0033-295X.108.2.393

[CR48] Logan, G. D., & Schneider, D. W. (2006). Priming or executive control? Associative priming of cue encoding increases “switch costs” in the explicit task-cuing procedure. *Memory & Cognition, 34*(6), 1250–1259. 17225506 10.3758/bf03193269

[CR49] Luria, R., & Meiran, N. (2003). Online order control in the psychological refractory period paradigm. *Journal of Experimental Psychology: Human Perception and Performance,**29*(3), 556–574. 10.1037/0096-1523.29.3.556 12848326 10.1037/0096-1523.29.3.556

[CR50] Luria, R., & Meiran, N. (2006). Dual route for subtask order control: Evidence from the psychological refractory paradigm. *The Quarterly Journal of Experimental Psychology, 59*(4), 720–744. 10.1080/02724980543000060 10.1080/02724980543000060 16707359

[CR51] Maquestiaux, F., Hartley, A. A., & Bertsch, J. (2004). Can practice overcome age-related differences in the psychological refractory period effect? *Psychology and Aging,**19*(4), 649–667. 10.1037/0882-7974.19.4.649 15584790 10.1037/0882-7974.19.4.649

[CR52] Mayr, U., & Awh, E. (2009). The elusive link between conflict and conflict adaptation. *Psychological Research, 73*(6), 794–802. 19034501 10.1007/s00426-008-0191-1 PMC4476291

[CR53] Meyer, D. E., & Kieras, D. E. (1997). A computational theory of executive cognitive processes and multiple-task performance: Part 2. Accounts of psychological refractory-period phenomena. *Psychological Review,**104*(4), 749–791. 10.1037/0033-295X.104.4.749 10.1037/0033-295X.104.4.749 9009880

[CR54] Miller, G. A. (1956). The magical number seven, plus or minus two: Some limits on our capacity for processing information. *Psychological Review, 63*(2), 81–97. 13310704

[CR55] Miller, J., & Durst, M. A. (2015). Comparison of the psychological refractory period and prioritized processing paradigms: Can the response-selection bottleneck model explain them both? *Journal of Experimental Psychology: Human Perception and Performance, 41*(5), 1420–1441. 26168143 10.1037/xhp0000103

[CR56] Miller, J., Ulrich, R., & Rolke, B. (2009). On the optimality of serial and parallel processing in the psychological refractory period paradigm: Effects of the distribution of stimulus onset asynchronies. *Cognitive Psychology, 58*(3), 273–310. 19281972 10.1016/j.cogpsych.2006.08.003

[CR57] Mittelstädt, V., & Miller, J. (2017). Separating limits on preparation versus online processing paradigms: Evidence for resource models. *Journal of Experimental Psychology: Human Perception and Performance, 43*(1), 89–102. 27808552 10.1037/xhp0000277

[CR58] Miyake, A., Friedman, N. P., Emerson, M. J., Witzki, A. H., Howerter, A., & Wager, T. D. (2000). The unity and diversity of executive functions and their contributions to complex “frontal lobe” tasks: A latent variable analysis. *Cognitive Psychology, 41*(1), 49–100. 10945922 10.1006/cogp.1999.0734

[CR59] Muhle-Karbe, P. S., Jiang, J., & Egner, T. (2018). Causal evidence for learning-dependent frontal lobe contributions to cognitive control. *Journal of Neuroscience, 38*(4), 962–973. 29229706 10.1523/JNEUROSCI.1467-17.2017 PMC5783969

[CR60] Musslick, S., & Cohen, J. D. (2021). Rationalizing constraints on the capacity for cognitive control. *Trends in Cognitive Sciences, 25*(9), 757–775. 34332856 10.1016/j.tics.2021.06.001

[CR61] Nino, R. S., & Rickard, T. C. (2003). Practice effects on two memory retrievals from a single cue. *Journal of Experimental Psychology: Learning, Memory, and Cognition, 29*(3), 373–388. 12776748 10.1037/0278-7393.29.3.373

[CR62] Orscheschek, F., Strobach, T., Schubert, T., & Rickard, T. (2019). Two retrievals from a single cue: A bottleneck persists across episodic and semantic memory. *Quarterly Journal of Experimental Psychology, 72*(5), 1005–1028. 10.1177/1747021818776818 29703125

[CR63] Otermans, P. C., Parton, A., & Szameitat, A. J. (2022). The working memory costs of a central attentional bottleneck in multitasking. *Psychological Research, 86*, 1774–1791. 34751812 10.1007/s00426-021-01615-1 PMC9363301

[CR64] Pashler, H. (1994). Dual-task interference in simple tasks: Data and theory. *Psychological Bulletin,**116*(2), 220–244. 10.1037/0033-2909.116.2.220 7972591 10.1037/0033-2909.116.2.220

[CR65] Pashler, H., & Johnston, J. C. (1989). Chronometric evidence for central postponement in temporally overlapping tasks. *The quarterly journal of experimental psychology a: Human*. *Experimental Psychology, 41*(1-A), 19–45.

[CR66] Pashler, H., & Johnston, J. C. (1998). Attentional limitations in dual-task performance. In H. Pashler (Ed.), *Attention*. Taylor & Francis.

[CR67] Ruiz Fernández, S., Leonhard, T., Rolke, B., & Ulrich, R. (2011). Processing two tasks with varying task order: Central stage duration influences central processing order. *Acta Psychologica, 137*(1), 10–17. 21427007 10.1016/j.actpsy.2011.01.016

[CR68] Ruthruff, E., Johnston, J. C., & Van Selst, M. (2001). Why practice reduces dual-task interference. *Journal of Experimental Psychology: Human Perception and Performance, 27*(1), 3. 11248938

[CR69] Ruthruff, E., Johnston, J. C., Van Selst, M., Whitsell, S., & Remington, R. (2003). Vanishing dual-task interference after practice: Has the bottleneck been eliminated or is it merely latent? *Journal of Experimental Psychology: Human Perception and Performance, 29*(2), 280. 12760615 10.1037/0096-1523.29.2.280

[CR70] Ruthruff, E., Van Selst, M., Johnston, J. C., & Remington, R. (2006). How does practice reduce dual-task interference: Integration, automatization, or just stage-shortening? *Psychological Research,**70*(2), 125–142. 10.1007/s00426-004-0192-7 16703392 10.1007/s00426-004-0192-7

[CR71] Salvucci, D. D., & Taatgen, N. A. (2008). Threaded cognition: An integrated theory of concurrent multitasking. *Psychological Review,**115*(1), 101–130. 10.1037/0033-295X.115.1.101 18211187 10.1037/0033-295X.115.1.101

[CR72] Sangals, J., Wilwer, M., & Sommer, W. (2007). Localizing practice effects in dual-task performance. *Quarterly Journal of Experimental Psychology, 60*(6), 860–876. 10.1080/17470210600822720 17514598

[CR73] Schmidt, J. R. (2019). Evidence against conflict monitoring and adaptation: An updated review. *Psychonomic Bulletin & Review, 26*, 753–771. 30511233 10.3758/s13423-018-1520-z

[CR74] Schmidt, J. R., Liefooghe, B., & De Houwer, J. (2020). An episodic model of task switching effects: Erasing the homunculus from memory. *Journal of Cognition, 3*(1), 22. 32964181 10.5334/joc.97 PMC7485406

[CR75] Schubert, T. (2008). The central attentional limitation and executive control. *Frontiers in Bioscience, 13*(13), 3569–3580. 18508456 10.2741/2950

[CR76] Schubert, T., Liepelt, R., Kübler, S., & Strobach, T. (2017). Transferability of dual-task coordination skills after practice with changing component tasks. *Frontiers in Psychology, 8*, 956. 28659844 10.3389/fpsyg.2017.00956 PMC5468462

[CR77] Schubert, T., & Strobach, T. (2018). Practice-related optimization of dual-task performance: Efficient task instantiation during overlapping task processing. *Journal of Experimental Psychology: Human Perception and Performance,**44*(12), 1884–1904. 10.1037/xhp0000576 30335413 10.1037/xhp0000576

[CR78] Schumacher, E. H., Seymour, T. L., Glass, J. M., Fencsik, D. E., Lauber, E. J., Kieras, D. E., & Meyer, D. E. (2001). Virtually perfect time sharing in dual-task performance: Uncorking the central cognitive bottleneck. *Psychological Science, 12*(2), 101–108. 11340917 10.1111/1467-9280.00318

[CR79] Shenhav, A., Botvinick, M. M., & Cohen, J. D. (2013). The expected value of control: An integrative theory of anterior cingulate cortex function. *Neuron, 79*(2), 217–240. 23889930 10.1016/j.neuron.2013.07.007 PMC3767969

[CR80] Sigman, M., & Dehaene, S. (2006). Dynamics of the central bottleneck: Dual-task and task uncertainty. *PLoS Biology, 4, Article e220*. 10.1371/journal.pbio.0040220 PMC1481521 16787105

[CR81] Simon, J. R., & Small, A., Jr. (1969). Processing auditory information: Interference from an irrelevant cue. *Journal of Applied Psychology, 53*(5), 433–435. 5366316 10.1037/h0028034

[CR82] Sinnett, S., Spence, C., & Soto-Faraco, S. (2007). Visual dominance and attention: The Colavita effect revisited. *Perception & Psychophysics, 69*(5), 673–686. 17929691 10.3758/bf03193770

[CR83] Strobach, T. (2020). The dual-task practice advantage: Empirical evidence and cognitive mechanisms. *Psychonomic Bulletin & Review,**27*(1), 3–14. 10.3758/s13423-019-01619-4 31152433 10.3758/s13423-019-01619-4

[CR84] Strobach, T., Frensch, P. A., & Schubert, T. (2012a). Video game practice optimizes executive control skills in dual-task and task switching situations. *Acta Psychologica, 140*(1), 13–24. 22426427 10.1016/j.actpsy.2012.02.001

[CR85] Strobach, T., Frensch, P. A., Soutschek, A., & Schubert, T. (2012b). Investigation on the improvement and transfer of dual-task coordination skills. *Psychological Research, 76*(6), 794–811. 21947746 10.1007/s00426-011-0381-0

[CR86] Strobach, T., Frensch, P. A., Müller, H. J., & Schubert, T. (2012c). Age- and practice-related influences on dual-task costs and compensation mechanisms under optimal conditions of dual-task performance. *Aging, Neuropsychology, and Cognition, 19*(1/2), 222–247. 10.1080/13825585.2011.630973 22168474

[CR87] Strobach, T., Frensch, P., Müller, H. J., & Schubert, T. (2012d). Testing the limits of optimizing dual-task performance in younger and older adults. *Frontiers in Human Neuroscience, 6*, 39. 10.3389/fnhum.2012.00039 PMC3293297 22408613

[CR88] Strobach, T., Hendrich, E., Kübler, S., Müller, H., & Schubert, T. (2018). Processing order in dual-task situations: The “first-come, first-served” principle and the impact of task order instructions. *Attention, Perception, & Psychophysics, 80*(7), 1785–1803. 10.3758/s13414-018-1541-8 29978280

[CR89] Strobach, T., Kübler, S., & Schubert, T. (2021). A Gratton-like effect concerning task order in dual-task situations. *Acta Psychologica, 217, Article 103328*. 10.1016/j.actpsy.2021.103328 10.1016/j.actpsy.2021.103328 33991794

[CR90] Strobach, T., Kürten, J., & Huestegge, L. (2023). Benefits of repeated alternations—Task-specific vs. task-general sequential adjustments of dual-task order control. *Acta Psychologica, 236*, Article 103921. 10.1016/j.actpsy.2023.103921 10.1016/j.actpsy.2023.103921 37084474

[CR91] Strobach, T., Liepelt, R., Pashler, H., Frensch, P. A., & Schubert, T. (2013). Effects of extensive dual-task practice on processing stages in simultaneous choice tasks. *Attention, Perception, & Psychophysics,**75*, 900–920. 10.3758/s13414-013-0451-z 10.3758/s13414-013-0451-z 23580330

[CR92] Strobach, T., Salminen, T., Karbach, J., & Schubert, T. (2014a). Practice-related optimization and transfer of executive functions: A general review and a specific realization of their mechanisms in dual tasks. *Psychological Research, 78*(6), 836–851. 24668506 10.1007/s00426-014-0563-7

[CR93] Strobach, T., Schubert, T., Pashler, H., & Rickard, T. (2014b). The specificity of learned parallelism in dual-memory retrieval. *Memory & Cognition, 42*, 552–569. 24170418 10.3758/s13421-013-0382-x

[CR94] Strobach, T., Schütz, A., & Schubert, T. (2015). On the importance of task I and error performance measures in PRP dual-task studies. *Frontiers in Psychology, 6*, 403. 25904890 10.3389/fpsyg.2015.00403 PMC4387374

[CR95] Strobach, T., & Wendt, M. (2022). Trial-to-trial modulation of task-order switch costs survive long intertrial intervals. *BMC Psychology,**10*(1), 77. 10.1186/s40359-022-00784-x 35317848 10.1186/s40359-022-00784-x PMC8941775

[CR96] Strobach, T., Wendt, M., Tomat, M., Luna-Rodriguez, A., & Jacobsen, T. (2020). No evidence for the reduction of task competition and attentional adjustment during task-switching practice. *Acta Psychologica,**204*, 103036. 10.1016/j.actpsy.2020.103036 32086004 10.1016/j.actpsy.2020.103036

[CR97] Stroop, J. R. (1935). Studies of interference in serial verbal reactions. *Journal of Experimental Psychology, 18*(6), 643–662.

[CR98] Szameitat, A. J., Lepsien, J., von Cramon, D. Y., Sterr, A., & Schubert, T. (2006). Task-order coordination in dual-task performance and the lateral prefrontal cortex: An event-related fMRI study. *Psychological Research,**70*(6), 541–552. 10.1007/s00426-005-0015-5 16142491 10.1007/s00426-005-0015-5

[CR99] Szameitat, A. J., Schubert, T., Müller, K., & von Cramon, D. Y. (2002). Localization of executive functions in dual-task performance with fMRI. *Journal of Cognitive Neuroscience, 14*(8), 1184–1199. 12495525 10.1162/089892902760807195

[CR100] Taatgen, N. A., & Lee, F. J. (2003). Production compilation: A simple mechanism to model complex skill acquisition. *Human Factors, 45*(1), 61–76. 12916582 10.1518/hfes.45.1.61.27224

[CR101] Töllner, T., Strobach, T., Schubert, T., & Müller, H. J. (2012). The effect of task order predictability in audio-visual dual task performance: Just a central capacity limitation? *Frontiers in Integrative Neuroscience, 6*, 75. 22973208 10.3389/fnint.2012.00075 PMC3438480

[CR102] Tombu, M., & Jolicœur, P. (2004). Virtually no evidence for virtually perfect time-sharing. *Journal of Experimental Psychology: Human Perception and Performance, 30*(5), 795–810. 15462621 10.1037/0096-1523.30.5.795

[CR103] Van Selst, M., Ruthruff, E., & Johnston, J. C. (1999). Can practice eliminate the psychological refractory period effect? *Journal of Experimental Psychology: Human Perception and Performance, 25*(5), 1268–1283. 10531663 10.1037//0096-1523.25.5.1268

[CR104] Welford, A. T. (1952). The ‘psychological refractory period’and the timing of high-speed performance—A review and a theory. *British Journal of Psychology. General Section, 43*(1), 2–19.

[CR105] Welford, A. T. (1980). The single-channel hypothesis. In A. T. Welford (Ed.), *Reaction times* (pp. 215–252). Academic Press.

